# Differential Group Delay of the Frequency Following Response Measured Vertically and Horizontally

**DOI:** 10.1007/s10162-016-0556-x

**Published:** 2016-02-26

**Authors:** Andrew King, Kathryn Hopkins, Christopher J. Plack

**Affiliations:** School of Psychological Sciences, University of Manchester, Manchester Academic Health Science Centre, Manchester, Greater Manchester M13 9PL UK

**Keywords:** frequency following response, phase locking, group delay, electroencephalography, temporal fine structure, temporal envelope

## Abstract

The frequency following response (FFR) arises from the sustained neural activity of a population of neurons that are phase locked to periodic acoustic stimuli. Determining the source of the FFR noninvasively may be useful for understanding the function of phase locking in the auditory pathway to the temporal envelope and fine structure of sounds. The current study compared the FFR recorded with a horizontally aligned (mastoid-to-mastoid) electrode montage and a vertically aligned (forehead-to-neck) electrode montage. Unlike previous studies, envelope and fine structure latencies were derived simultaneously from the same narrowband stimuli to minimize differences in cochlear delay. Stimuli were five amplitude-modulated tones centered at 576 Hz, each with a different modulation rate, resulting in different side-band frequencies across stimulus conditions. Changes in response phase across modulation frequency and side-band frequency (group delay) were used to determine the latency of the FFR reflecting phase locking to the envelope and temporal fine structure, respectively. For the FFR reflecting phase locking to the temporal fine structure, the horizontal montage had a shorter group delay than the vertical montage, suggesting an earlier generation source within the auditory pathway. For the FFR reflecting phase locking to the envelope, group delay was longer than that for the fine structure FFR, and no significant difference in group delay was found between montages. However, it is possible that multiple sources of FFR (including the cochlear microphonic) were recorded by each montage, complicating interpretations of the group delay.

## Introduction

Neural firing in the auditory system can phase lock to the filtered output of the basilar membrane (Tasaki^1954^; Rose et al. 1967; Smith et al. 1975; Palmer and Russell 1986). Phase locking is important for a precise temporal code integral to pitch perception (e.g., Cariani and Delgutte 1996; Krishnan and Plack 2011), localization (Rose et al. 1966), and speech perception (Møller 1999; Krishnan et al. 2004, 2005) particularly in noisy environments and adverse listening conditions (Sachs et al. 1983).

In animal models, phase locking has been measured directly in neurons throughout the subcortical afferent auditory pathway, from the auditory nerve (Tasaki 1954) and cochlear nucleus (Galambos and Davis 1943) up to at least the inferior colliculus (Rose et al. 1966; Smith et al. 1975). In humans, consistent phase locking to periodicites in acoustic stimuli can be measured at the scalp (using electroencephalography), as the frequency following response (FFR) (Worden and Marsh 1968; Moushegian et al. 1973; Glaser et al. 1976; Stillman et al. 1976). At the scalp, it is difficult to determine which neural structures generate the FFR. Early reports assumed the FFR emanates from a single source (Gerken et al. 1975; Smith et al. 1975). However, later studies showed that multiple sources are measurable under certain recording conditions (Stillman et al. 1978; Gardi et al. 1979; Batra et al. 1986; Galbraith 1994; Galbraith et al. 2000; Galbraith et al. 2001; Bidelman 2015).

Vertical and horizontal electrode montage orientations differentially emphasize later and earlier (respectively) Jewett Waves (Jewett and Williston 1971) in click-evoked auditory brainstem responses (ABRs) (Picton et al. 1974; Scherg and Von Cramon 1985; Galbraith 1994; Parthasarathy and Bartlett 2012). Likewise, differently oriented montages may differentiate FFR sources also (Stillman et al. 1978; Scherg and Brinkmann 1979; Galbraith 1994; Galbraith et al. 2000; Galbraith et al. 2001). Stillman et al. (1978) recorded FFRs to pure-tone stimuli (167 to 500 Hz) from vertex to earlobe (vertical montage) and from earlobe to earlobe (horizontal montage). The FFR waveforms were complex, with two peaks per stimulus period. In the horizontal montage, the leading peaks were prominent; in the vertical montage, the lagging peaks were prominent. The time between the leading and lagging peaks was 1.7 ms, regardless of frequency or level, reflecting a putative phase shift between two sources of FFR. From this, Stillman et al. (1978) determined that horizontal and vertical montages emphasized shorter and longer latency FFR, respectively.

Visual inspection to determine onset latency is subjective and thus not easily defined or repeated. More quantifiable methods of determining generation sites of the FFR exist, such as inferring a source from the overall response latency. Methods to do this include group delay (e.g., Batra et al. 1986) and cross-correlation between stimulus and response (Galbraith 1994). Galbraith (1994) found that a 200-Hz pure-tone stimulus had a maximum cross-correlation latency equivalent to the latency expected from an auditory nerve source for the horizontally recorded FFR, whereas for the vertically recorded FFR, the latency suggested a source in the lateral lemniscus. The correspondence of latency to anatomical structure was based in previous dipole modeling of ABRs by Scherg and Von Cramon (1985). FFRs to the missing fundamental (200 Hz) of a harmonic stimulus (sum of 600, 800, and 1000 Hz tones) were often not present or very weak in the horizontal record but were well recorded by the vertical record and had a maximum cross-correlation latency that again suggested generation in the lateral lemniscus. However, taking the latency of the maximal cross-correlation between stimulus and response is ambiguous for latencies greater than the stimulus periodicity (restricting its usefulness to low-frequency stimuli).

Group delay is the derivative (slope) of the phase response with respect to frequency. For a given delay, higher frequency sinusoids will have a larger unwrapped phase angle than lower frequency sinusoids. Provided the delay is less than the reciprocal of the frequency spacing between consecutive sinusoids, their phases will be less than one cycle apart. Then, the phase response can be unambiguously unwrapped to determine the latency.

Batra et al. (1986) determined group delay from the phase of the Fourier transforms of FFRs to pure tones of various frequencies. At low frequencies, there appeared to be a steeper change in phase with frequency (larger group delay) than at higher frequencies. For tones below about 300 Hz, group delay was about 8 ms. Above 1000 Hz, group delay was less than 1 ms. Batra et al. (1986) suggested that the change in group delay from low to high frequency indicates different generation sites but that the cochlear microphonic (CM) was responsible for the shorter group delay. The CM is the nonneural electrical potential generated by the cochlear hair cells that mimics the stimulus (Terkildsen et al. 1974).

Batra et al. (1986) used pure tones to evoke FFRs; they did not investigate FFR to the temporal envelope of complex stimuli. Galbraith (1994) used a harmonic stimulus to generate FFR that followed the missing fundamental frequency (i.e., the envelope) of the stimulus and measured latency using cross-correlation rather than group delay. In addition, he did not consider the FFR to the temporal fine structure (TFS) of this complex stimulus, instead comparing envelope-FFR to pure-tone FFR at the same frequency. In other words, the stimuli would have had separate cochlear representations, with the pure tone exciting more apical sites which are subject to a larger delay in the cochlear travelling wave (Ruggero and Temchin 2007). The current study used group delay to investigate the latency of FFR to the TFS and to the temporal envelope of the *same stimuli*, with differential electrode configurations. This should help to minimize differences due to cochlear delay, assuming that envelope- and TFS-FFR for these stimuli are generated by the same region of the cochlea. This is a plausible assumption because differences in FFR with changes in the cut-off of high-pass masking noise suggest that FFRs to low frequency tones at high sound levels are generated by the low frequency skirt of a broad area of stimulation across the apical half of the basilar membrane (Gardi and Merzenich 1979). Horizontally and vertically aligned electrode montages were used to determine if distinct earlier and later sources were observed for FFR to the two temporal properties of a series of amplitude-modulated tones.

## Materials and Methods

### Listeners

Twelve male and 11 female listeners (18 to 31 years, mean = 23 years) with audiometric thresholds below 25-dB hearing level at 0.25, 0.5, 1.24, and 8 kHz were recruited. All procedures of the study were approved by the Research Ethics Committee at the University of Manchester.

### Stimuli

**FIG. 1:**
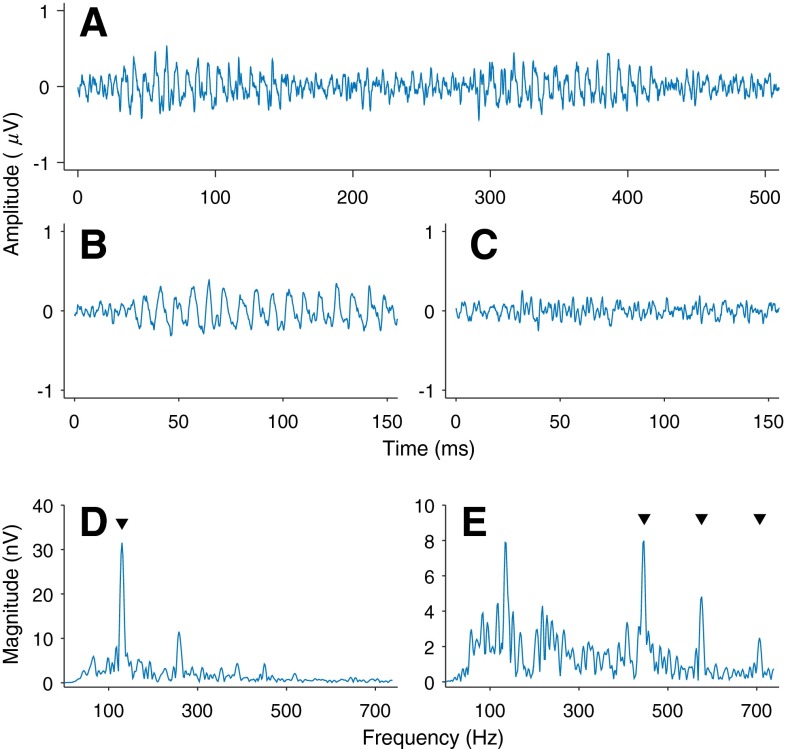
The grand mean (across listeners and trials) frequency following response (FFR) to the alternating polarity presentation of one stimulus condition (*f*_*m*_ = 130 Hz). **A** The mean FFR waveform for both polarities in sequence. **B** Addition of the responses to the two polarities. **C** Subtraction of the second response from the first. **D** The Fast Fourier transform power spectrum of the addition waveform with a downward arrow denoting *f*_*m*_. **E** The power spectrum of the subtraction waveform with *downward arrows* denoting the three component frequencies.

Stimuli consisted of three equal-amplitude pure tones summed together. The highest and lowest tone frequencies were equidistant from the center tone frequency, thus creating an amplitude-modulated tone with a modulation rate (*f*_*m*_) equal to the frequency spacing. Five frequency spacings were used, all with the same center tone frequency of 576 Hz (see Table 1). This created three frequency regions that could be used to estimate group delay: the *f*_*m*_, the frequency of the lower side-tone, and the frequency of the higher side-tone (far left, middle left, and far right columns of Table 1, respectively). The FFR was analyzed in each of these frequency regions. A center frequency of 576 Hz was used because around 500 Hz, FFR is typically stronger than at lower or higher frequencies (Glaser et al. 1976). 576 Hz was selected to avoid harmonics of the 50-Hz electrical mains/ground artifact. Modulation rates ranging from 85 to 145 Hz were used to strike a balance between being sufficiently high as to limit cortical contributions to the FFR (Herdman et al. 2002; Joris et al. 2004), while also being low enough to elicit envelope-FFRs (envelope-FFR amplitude decreases with increasing *f*_*m*_; Purcell et al. 2004; Parthasarathy and Bartlett 2012). All stimuli were presented at a root mean square average of 85 dB SPL. For each trial, the modulated tones were presented first for 140 ms with all three sinusoidal components starting in sine phase (positive starting polarity), then after a 120-ms silent interval, presented again for 140 ms with all three sinusoidal components starting with π radian phase (antiphase, or negative starting polarity). Another silent interval of 170 ms followed before the next presentation pair. Each 140-ms tone included 20-ms raised-cosine onset and offset ramps. When the response to the negative polarity stimulus is subtracted from the response to the positive polarity stimulus, the response following the TFS adds constructively and the response following the envelope mostly cancels out (Goblick and Pfeiffer 1969). This subtraction waveform was used to quantify the TFS-FFR, which was analyzed over the lower and higher side-tone frequency regions. On the other hand, when the responses to the two stimuli of opposing polarities are summed, the response following the TFS mostly cancels out, whereas the response following the envelope adds constructively (see Aiken and Picton 2008). This addition waveform was used to quantify the envelope-FFR, which was analyzed over the modulation frequency (*f*_*m*_) region. These two manipulations are shown in Figure 1 for the grand mean across listeners and trials in the condition with *f*_*m*_ = 130 Hz. The amplitudes of the addition and subtraction waveforms were halved to account for the effective doubling of trials by combining the responses to the two stimulus polarities.

**TABLE 1:** The frequency components (in hertz) of the five stimuli over which group delay were calculated

Modulation	Lower side-tone	Center frequency	Upper side-tone
85	491	576	661
100	476	576	676
115	461	576	691
130	446	576	706
145	431	576	721

Each row corresponds to one stimulus

**FIG. 2:**
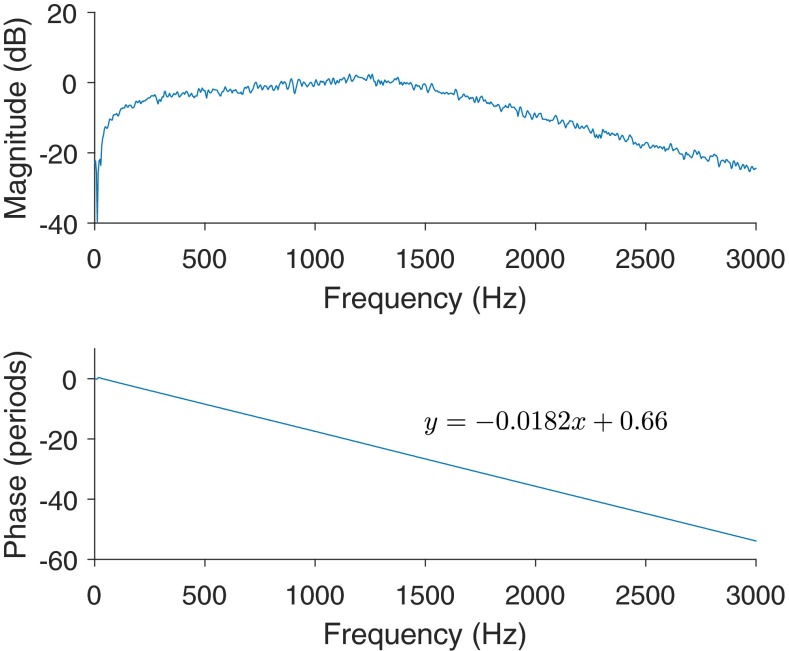
The magnitude (*top panel*) and phase (*bottom panel*) spectra of the tubing and earphone frequency response, measured relative to a broad-band Gaussian noise output generated by the same frequency analyzer. The *inset equation in the bottom panel* describes the best linear fit for the phase response. The slope (−0.0182) suggests a group delay of 18.2 ms.

The five stimuli were tested separately in blocks of 1600 trials (producing 3200 responses as each trial elicited two responses, one to the sine-phase stimulus and one to the antiphase stimulus). Responses were stored for analysis as 16 subaverages (each an average of 100 trials). Only subaverages were stored for analysis. The stimuli were created in MATLAB (The Mathworks, Massachusetts) and presented using Tucker Davis Technologies (TDT, Florida) SigGen and BioSigRP software. Stimuli were converted to analogue signals by a TDT RP2.1 processor and transduced to acoustic waves outside the listening booth by an ER30 earphone (Etymotic Research, Illinois) to minimize stimulus artifact. The transducer was connected to the listener by 6 m of tubing terminating in the listener’s right external auditory canal through a foam earplug. The frequency response of the transducer and tubing was measured by playing white noise, from a spectrum analyzer, through the transducer and tubing into an IEC711 2-cc coupler which input back into the analyzer. The magnitude spectrum was flat until about 1.5 kHz, rolling off at 20 dB/oct above that; the phase spectrum had a linear slope with respect to frequency. This slope suggested a group delay of 18.2 ms. These spectra are seen in Figure 2. The listener’s left ear was plugged with a foam plug. Pilot tests showed that no stimulus artifact was recorded when the stimulus was presented with the tubing detached from the earplug and sealed with tape.

The electrical field potential was recorded by two montages of gold-plated passive electrodes. The horizontal montage recorded at the ipsilateral mastoid (referenced to the contralateral mastoid) and the vertical montage recorded at the seventh cervical vertebra (referenced to the forehead hairline on the sagittal line). Both montages shared a common ground electrode on the listener’s brow. The electrodes were wired into a TDT RA16LI-D head-stage linked to a TDT RA4PA preamplifier and analogue-to-digital convertor. The signal was converted at a sample rate of 24,414 Hz. The digital signal was sent via fiber optics to a TDT RA16 Medusa Base Station for processing. The base station and RP2.1 processor were linked for clock synchronization and both communicated with the BioSigRP software via optical fibers. No online filtering was applied to the recordings. Individual trials with peak amplitude exceeding ±60 μV were rejected from the subaverages. Listeners lay in a reclining chair and were asked to relax as much as possible and try to sleep during the recordings. Listener wakefulness was not recorded.

### Analysis

Recordings were exported to text files, read and analyzed by MATLAB scripts. Records were divided into the horizontal and vertical montages. All subaverages were digitally filtered by a fourth-order Butterworth band-pass filter between 60 and 2000 Hz. The filtering was zero-phase because it was implemented in both forward and reverse directions across time using the “filtfilt” MATLAB function. This doubles the filter order to an eighth-order Butterworth band-pass filter. After this filtering, any subaverages with peak amplitude exceeding ±35 μV were removed before further averaging. For the envelope-FFR, the magnitude of the discrete Fourier transform (DFT) at *f*_*m*_ was calculated from the mean addition waveform for each stimulus condition. For the TFS-FFR, magnitude of the DFT at the lower side-tone and upper side-tone frequencies was calculated from the mean from the subtraction waveform.

**FIG. 3:**
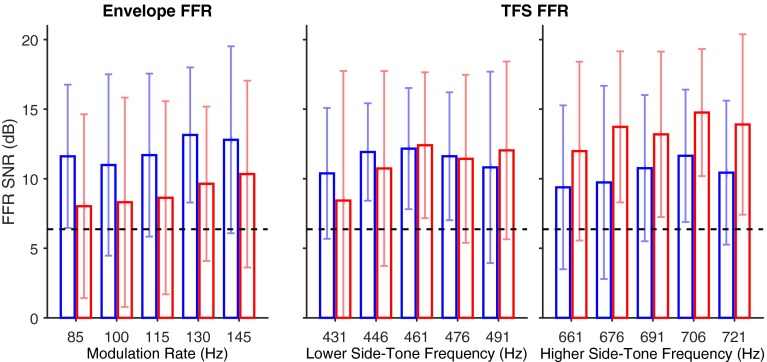
Averaged across listeners with standard deviation error bars, the envelope-FFR signal-to-noise ratio (SNR) across *f*_*m*_ is given in the *left panel* and TFS-FFR SNR across the lower side-tones and the higher side-tones are given in the *middle* and *right panels*, respectively. In each panel, the FFR from the vertical montage is in *blue*, the FFR from the horizontal montage is in *red*, and the SNR criterion of 5.3 dB is given by the *black dashed line*.

A statistical criterion based on the signal-to-noise power ratio (SNR) was used to determine the presence or absence of a response to the stimulus. The signal power was taken as the DFT power at the FFR frequency. The noise power was taken as the square of the mean magnitude across frequencies, selected at a resolution of 4 Hz, from 9 to 37 Hz above and below the FFR frequency. FFR was accepted as present if the F-ratio of the signal power (two degrees of freedom) over the noise power (32 degrees of freedom) was less than 1 % likely given the null hypothesis that signal and noise power are the same, using the F inverse cumulative distribution function (Dobie and Wilson 1996). The phase of the DFT was used to calculate group delay only when the FFR magnitude passed this criterion. The mean SNR of FFR, across listeners, at each frequency is shown in Figure 3. The standard deviation in FFR SNR shows that it was not always sufficiently above the noise floor to pass the SNR criterion. The phase angles of the FFR were unwrapped for each frequency region (modulation rates, lower side-tones, upper side-tones, see Table 1). Before unwrapping the phase, the 18.2-ms group delay created by the tubing (between the transducer and listener’s ear) was corrected for. This was done by adding 18.2e^−3^*f* – *floor*(18.2e^−3^*f*) to the phase of the DFT. The *floor*(18.2e^−3^*f*) term was included to remove the redundant full phase cycles before unwrapping but did not affect following group delay calculation. Group delay for a frequency region was calculated only if the FFR at three or more frequencies in that frequency region passed the SNR criterion.

### Phase Unwrapping

**FIG. 4:**
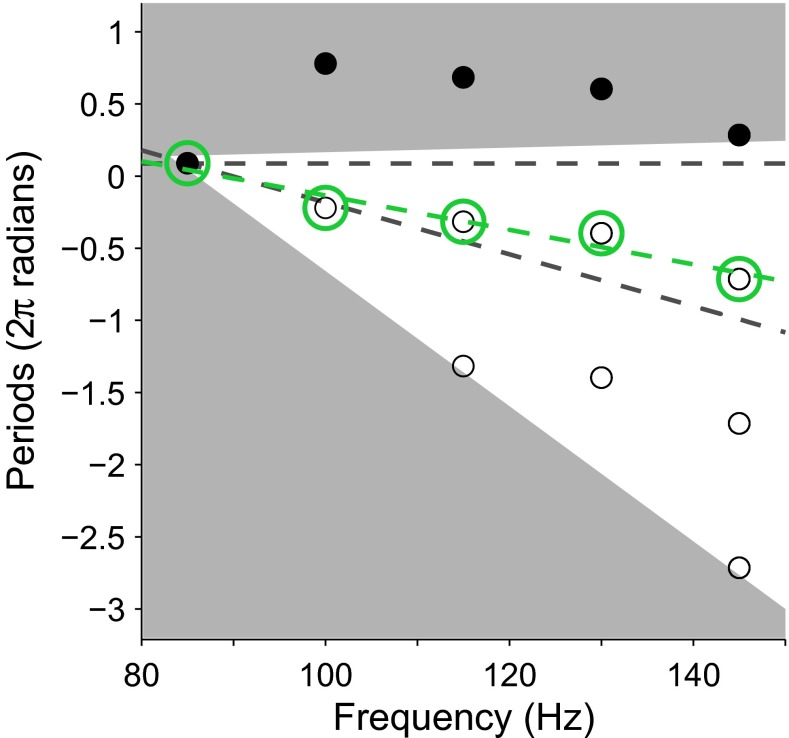
A model example of the phase unwrapping process. The initial phase values of the DFT at each modulation frequency (*filled black circles*) are outside the limits of unwrapping possibilities for feasible group delays (*gray area*). Only phase values within the white area were considered by the unwrapping algorithm. The *open circles* indicate unwrapping possibilities considered by the unwrapping algorithm, in this example. The *green circles* indicate the unwrapping option that provides the best linear fit (*green dashed line*) within the boundaries of the fits allowed by the algorithm (*black dashed lines*).

Because a group delay fit could be made with phase values at a minimum of three out of five frequency points in any given frequency region meeting the SNR criterion, gaps of 30 or 45 Hz between consecutive phase values occasionally existed. Without any missing phase values (a frequency spacing of 15 Hz), sequential unwrapping would be unambiguous for group delays under 33.3 ms, because a slope of −0.033 or less would be required for more than half a cycle to pass between phase values at frequencies 15 Hz apart. However, if phase values were missing at one or two consecutive frequency points (frequency spacing of 30 and 45 Hz respectively), the maximum group delay for which unambiguous unwrapping is possible dropped to 16.7 and 11.1 ms, respectively. As such group delays were within the test range, sequentially unwrapping the phase within tolerances of ±π sometimes led to poor linear fits or linear-fit slopes suggesting physiologically unreasonable latencies. Instead, all possible unwrapping possibilities that could produce group delays between 0 and 20 ms were calculated. In Figure 4, these group delay limits are shown by the two dashed black lines. These limits were used to avoid negative or very long group delay estimation, but they still allowed group delays indicative of CM responses or thalamo-cortical responses.

Using the lowest frequency phase value as an anchor, the other phase values were initially unwrapped to the nearest, but greater, phase value (which would produce a negative, and impossible, group delay). Then, integer periods were successively subtracted from the nonanchored phase values. All possible combinations of unwrapping from 0 periods to 0.04*Δx periods were calculated (for each frequency point, where *Δx* is the difference in frequency between the anchor and a given frequency point). This is shown by the nongray space in Figure 4. Each combination was fitted with a linear function but only fits with slopes between 0 and −0.02, and sum and squared residual errors less than 0.05 were accepted. Combinations outside the 0 to 20 ms boundaries were calculated to account for the possible residual error to the linear fit for each phase value. If one or more unwrapping combinations passed the fit criteria, the unwrapping that had the best linear fit was selected and the slope of that fit was taken as the group delay. Figure 4 shows an example of this method with model data.

## Results

As mentioned in the “Analysis” section, Figure 3 shows the mean and standard deviation (across listeners) of the SNR of the FFRs at the frequencies in each region. In the modulation frequency region, there is a trend for higher SNRs in the vertical montage than in the horizontal montage (left panel). In the lower side-tone frequency region the SNRs are similar in both montages (middle panel) and in the higher side-tone frequency region, the SNRs in the horizontal montage tend to be higher than in the vertical montage (right panel). This crossover interaction between montage and frequency region on FFR SNR (averaged across all five frequencies in each region) was found to be significant by repeated-measures ANOVA [*F*(2,44) = 17.75, *P* < 0.001].

**FIG. 5:**
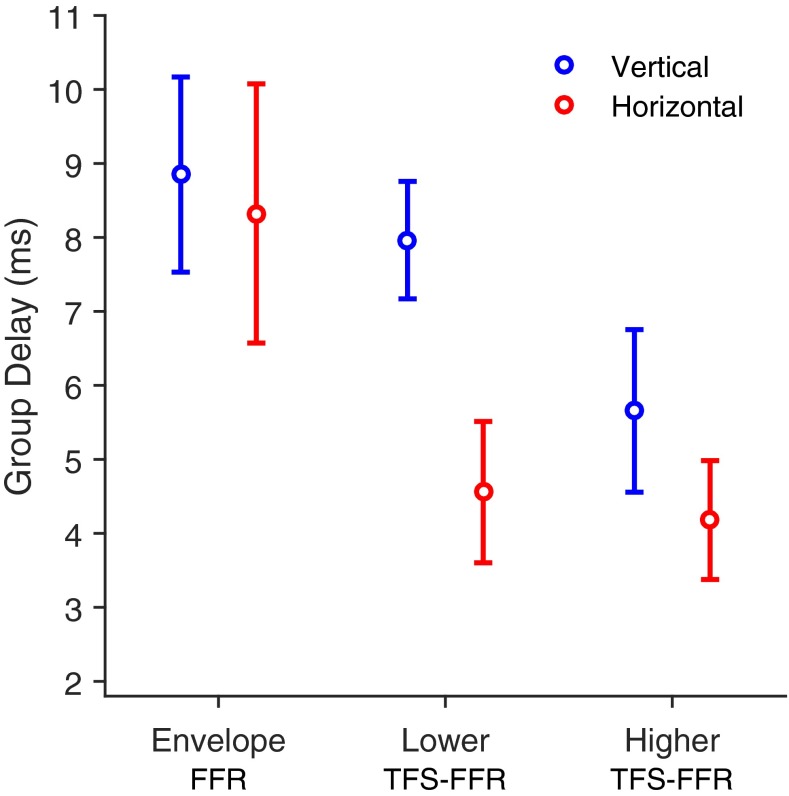
The mean group delays (across listeners) for the FFRs in the three frequency regions for the two montages. The frequency regions are the modulation rates for the envelope-FFR, the lower and higher side-tones for the TFS-FFR. The montages are vertical (*blue*) and horizontal (*red*). *Error bars* indicate confidence intervals of 95 %.

Envelope-FFR group delay was calculated across the modulation frequencies for each of the two montages (vertical and horizontal). For the TFS-FFR, two separate group delays were calculated for each montage, one for the lower side-tone frequencies, and one for the higher side-tone frequencies (see Table 1). For each listener, up to six group delays were calculated, but due to the imposition of an FFR SNR criterion and group delay slope and fit criteria, not all listeners’ data provided the full six group delay values. For each frequency region and montage, Table 2 shows the number of listeners for whom the FFR at each frequency point passed the SNR criterion. Table 2 also shows the number of listeners for whom at least three frequency points passed this criterion and produced a group delay that passed the slope and fit criteria. Twenty percent of the group delay data were missing. By frequency region, 17 % were missing from the modulation and lower side-tone regions, and 26 % were missing from the higher side-tone region. By montage, there were more missing group delays from the horizontal montage (27 %) than from the vertical montage (13 %). Of the group delays that passed the criteria, the mean group delay for each montage in each frequency region is plotted in Figure 5. The figure shows that the horizontal montage generally measured shorter group delays in the FFR than the vertical montage, most notably for the lower side-tone frequency region (and to a lesser extent the higher side-tone frequency region). However, the difference in group delay between the montages was small in the modulation frequency region.

**TABLE 2:** The number of FFR, recorded by each montage, that passed the criterion for on-frequency magnitude being sufficiently above noise floor, and the number of calculable group delays in each frequency region that passed the criterion for acceptable group delay

Frequency region	Modulation					Lower side-tone					Upper side-tone				
Frequency (Hz)	85	100	115	130	145	431	446	461	476	491	661	676	691	706	721
Vertical montage	19	17	21	22	21	18	19	20	20	20	16	17	16	19	17
Group delay	22					20					18				
Horizontal montage	16	14	17	16	18	16	17	18	17	18	17	20	19	22	19
Group delay	16					18					16

Fourteen listeners did not have a complete set of six group delays. In order to include the remaining group delay data from these listeners in further analysis, a linear mixed-effects model was used (“lmer” from the R package “lme4” version 1.1-9; Bates et al. 2015). This model allows unbalanced amounts of data across factor levels but assumes that the data are missing independent of the observed or missing data values. The interaction between the effects of montage and frequency region on group delay was analyzed as a fixed-effects factor. Listener identification number was included as a random-effect term to account for possible within-subject clustering effects (i.e., intrinsic listener effects). Estimation of the factor coefficients in the model was made by restricted maximum-likelihood estimation. An adjustment to the degrees of freedom was made to account for the small sample size (Kenward and Roger 1997). This was implemented using the “lmerTest” R package (Kuznetsova et al. 2015).

There was a statistically significant interaction between the effects of montage and frequency region on group delay [*F*(2,86) = 3.28, *P* = 0.042]. The random effect of listener was not significant [*χ*^2^(1) = 1.05, *P =* 0.300]. Differences between least-squares means from the model revealed that the group delay of the envelope-FFR in the modulation frequency region was not significantly different across montages [0.5 ms, *t*(83.8) = 0.64, *P =* 0.524]. For the TFS-FFR, the horizontal montage measured FFR with a significantly shorter group delay than the vertical montage for the lower side-tones [3.4 ms, *t*(84.4) = 4. 24, *P* < 0.001], but not for the higher side-tones [1.5 ms, *t*(82.8) = 1.79, *P* = 0.078]. This is seen in Figure 5.

Modulation-FFR group delay was significantly longer than the higher side-tone FFR group delay in both montages [3.2 and 4. 2 ms longer in vertical and horizontal montage, respectively, *t*(84) = 4.08, *t*(88.5) = 4.78, *P* < 0.001]. However, modulation-FFR group delay was only significantly longer than lower side-tone FFR group delay in the horizontal montage [3.7 ms, *t*(88) = 4.39, *P* < 0.001], not the vertical montage [0.8 ms, *t*(80.7) = 1.13, *P* = 0.262]. The lower and higher side-tone FFR group delay only differed significantly in the vertical montage [2.3 ms, *t*(85.8) = 2.92, *P* = 0.004], not the horizontal montage [0.4 ms, *t*(82.9) = 0.53, *P =* 0.596].

## Discussion

### Generation Sites of the FFR

For most listeners, group delays for the lower and higher side-tones of the TFS-FFR recorded with the horizontal montage were around 4 to 5 ms. Compared to the horizontal montage, the vertical montage recorded TFS-FFR with significantly longer lower side-tone group delays of about 8 ms, but only marginally longer higher side-tone group delays of about 6 ms. Both montages recorded modulation, or envelope, FFR with group delays around 8 to 9 ms. The difference between montages in lower side-tone TFS-FFR group delay supports previous claims that horizontal and vertical montages can record FFR from distinct earlier and later sources respectively (Stillman et al. 1978; Galbraith 1994). This finding may be limited to frequencies below the higher side-tone frequencies tested in the current study, possibly because phase locking in the rostral brainstem and midbrain is greatly reduced above around 500 to 1000 Hz (Liu et al. 2006). This is consistent with evidence that a vertical montage records more strongly low-pass filtered FFR than a horizontal montage (Galbraith et al. 2000; also see Figures 6 and 7 in Bidelman 2015), which Galbraith et al. considered as evidence that vertical montages measure rostral-brainstem generated FFR (i.e., later in the auditory pathway) and horizontal montages measure more caudal-brainstem generated FFR (i.e., earlier in the auditory pathway). In the current study, the mean FFR SNR in the horizontal montage was greater than in the vertical montage for the higher side-tone. It is likely that, at the higher side-tone frequency, the rostral brainstem does not contribute to the FFR as much as do more caudal generators. If the vertical montage is relatively insensitive to these caudal generators, this would explain the decrease in the FFR to the higher side-tone for the vertical montage compared to the horizontal montage. The switch to more caudal generation sites would also explain why the group delays in the vertical montage were shorter for the higher side-tone TFS-FFR than for the lower side-tone TFS-FFR.

It is possible that the envelope-FFR group delays did not differ significantly between montages because the FFRs are generated by the same source or sources. The mean envelope-FFR SNR across *f*_*m*_ (Figure 3, left panel) was greater for the vertical montage than for the horizontal montage, which suggests that the sources of envelope-FFR were better recorded by the vertical montage than the horizontal montage. That said, the horizontal montage successfully recorded a good proportion of the envelope-FFR, in contrast to Galbraith (1994), who found that envelope-FFRs were inconsistent and sometimes not present in the horizontal record. However, individual differences in group delay were large (standard deviations of the group delays were around 3.4 ms for modulation rates and around 2 ms for side-tones). It is unlikely that this was due to anatomical differences in the auditory pathway structure alone. The differences in group delay were likely to be due a complex interaction between position and orientation of FFR generators, electrode positions, and the strength of FFR from multiple generators, in addition to the inherent measurement error associated with measuring FFR through electroencephalography. For example, group delays longer than 12 ms for some listeners in the current study could possibly suggest contributions from thalamo-cortical regions (Kuwada et al. 2002).

The latencies of waves I to V of the ABR have been compared with the latencies of compound action potentials measured intracranially during surgical operations. Waves I and II are associated with the auditory nerve (Møller 2007), waves II and III originate from the rostral end of the auditory nerve or the cochlear nucleus with a latency of about 3 to 4 ms (Møller and Jannetta 1981, 1983), and wave V originates from the inferior colliculus with a latency of about 6 to 8 ms (Møller and Jannetta 1982; Møller et al. 1994). In the current study, the mean envelope-FFR group delays in both montages were consistent with latencies of wave V or later, suggesting sources in the rostral brainstem or midbrain, although with shorter latencies than the source modeled by Herdman et al. (2002) with auditory steady state responses to 88-Hz modulation of a 1-kHz tone. The lower side-tone TFS-FFR in the vertical montage appears to come from a source with a similar latency. The lower and higher side-tone TFS-FFRs in the horizontal montage appear to originate from the cochlear nucleus, while the higher side-tone TFS-FFR in the vertical montage appears to originate from somewhere between the cochlear nucleus and the inferior colliculus.

Parthasarathy and Bartlett (2012) measured envelope-FFRs and ABRs from rats using two electrode montages. One montage recorded ABRs with prominent wave III and no waves IV or V, suggesting cochlear nucleus generation. This montage also recorded envelope-FFRs that were stronger at higher *f*_*m*_ than lower *f*_*m*_. The second montage recorded ABRs with large waves I, IV, and V, linked to auditory nerve and inferior colliculus generation, and envelope-FFRs that were stronger to lower *f*_*m*_, which Parthasarathy and Bartlett (2012) argued were generated by the same generators as waves IV and V. Parthasarathy and Bartlett also showed that anesthesia reduced waves IV and V in the second montage, but not wave III in the first montage. In the first montage, anesthesia reduced 90- to 360-Hz envelope-FFRs, but in the second montage, anesthesia mostly reduced envelope-FFRs below 90 Hz. Overall, these results suggest that montages that measure ABR components linked to rostral brainstem generation will measure slower envelope-FFR best, while montages that measure more caudally generated waves of the ABR will measure faster rates of envelope-FFR. With respect to the FFR to pure tones, Galbraith et al. (2000) found that a horizontal montage recorded higher-frequency FFR better, while a vertical montage recorded lower-frequency FFR better. While Galbraith et al. (2000) and Parthasarathy and Bartlett (2012) only measured TFS-FFR and envelope-FFR, respectively, the current study considered both TFS and envelope-FFR for the same stimuli. The crossover interaction between montage and stimulus (pure-tone or modulation) frequency on FFR SNR is extended in the current study with an interaction between montage and FFR *type*: envelope-FFR versus higher side-one TFS-FFR.

Direct comparisons of ABR and FFR latencies may not be valid. Masking experiments have been used in an attempt to determine the cochlear region (from base to apex) in which the basilar membrane responds most in ABR (Don and Eggermont 1978) and FFR (Gardi and Merzenich 1979). While Gardi and Merzenich (1979) suggested that the FFR is generated from auditory nerve fibers synapsing in a fairly apical region of the cochlea (although not as apical as the place of exCitation), Don and Eggermont (1978) suggested that ABR waves II and III are dominated by contributions from the basal portions of the cochlea. Differences in the travelling wave delay between these cochlear regions should be taken into account. Other research supports the argument that ABR and FFR are not produced by the same mechanisms. Hoormann et al. (1992) found no correlations between FFR latencies (derived by cross-correlation) and the latencies of the click-evoked ABR waves. Bidelman (2015) found distinct differences between listeners’ FFRs to a click train, and the same click train convolved with their ABR waveform, suggesting that the FFR is not simply a series of overlapping ABR responses.

In the current study, although the acoustic delay due to the 6 m of tubing between the transducer and ear was taken into account before calculating group delay, the middle ear and cochlear travelling wave delays were not accounted for. Assuming that the stimuli in the current study excited the basilar membrane in a fairly apical region that has a characteristic frequency of 1 to 2 kHz (Gardi and Merzenich 1979), it is possible that the cochlear travelling wave delay was approximately 2 to 3 ms (Ruggero and Temchin 2007). Hence, it is possible that the TFS-FFR in the horizontal montage originated from the auditory nerve or could even be just the CM for some listeners. In the vertical montage, the TFS-FFR may have originated from the cochlear nucleus, inferior colliculus, or somewhere in between. Because the envelope- and TFS-FFR were derived from the same stimuli, the cochlear travelling wave delay should be similar for the two types of FFR. Therefore, differences in group delay between envelope- and TFS-FFR must be due to either differences in the dominant source or sources of phase locking to the envelope and TFS in the auditory pathway, or differences in contamination by the CM in the methods used to derive the envelope- and TFS-FFR (discussed below).

### Multiple Neural, and Microphonic, Sources Per Montage

A linear fit was used to determine the group delay in each frequency region in the current study. This assumes that the FFR group delay is constant in each frequency region for each montage. In some cases, group delay may not be constant across the frequency region due to multiple FFR sources with different frequency sensitivities, making a linear fit invalid. However, even if the group delay is constant across the frequency region, this does not rule out multiple sources. An arbitrary mix of sources with constant relative contributions to the FFR mix, as recorded by the electrode montage at the scalp, will also result in a constant group delay. Therefore it is not possible, using the methodology in the current study, to determine how many sources of FFR contributed to the signals recorded by each montage.

If two sources of FFR with differing latencies contributed equally (in amplitude) to the response, it would have a phase corresponding to the difference of the phases of the source FFRs. For example, the addition of a sine wave with phase 45° and a sine wave with phase 135° results in a sine wave with phase 90° and an amplitude larger by a factor of √2. However, this only holds for equal amplitude waves and a 90° phase difference. For differing amplitudes, the resultant wave’s phase is weighted toward the phase of the wave with the larger amplitude. Increasing the phase difference decreases the resultant wave’s amplitude until the two waves cancel completely at a phase difference of 180°. Batra et al. (1986) measured the FFRs to pure tones over a wide frequency range and found oscillations in spectral magnitude across frequency, which may be evidence of multiple FFR sources interacting destructively or constructively in the recorded response, and is dependent on stimulation frequency and latency between the sources.

Scherg and Von Cramon (1985) demonstrated that potentials recorded at the scalp are a composite of dipole sources within the brain. A dipole model of click ABR associated wave I with the auditory nerve but suggested that later waves were a more complex combination of dipoles from multiple structures including the lateral lemniscus, trapezoid body, and inferior colliculus (Scherg and Von Cramon 1985). Herdman et al (2002) and Bidelman (2015) also used dipole modeling with 46 or 64 electrodes, respectively, distributed across the scalp to determine the sources of sustained responses such as FFR. Herdman et al. (2002) measured envelope FFR to a 1-kHz tone sinusoidally amplitude-modulated at 12, 39, and 88 Hz, while Bidelman (2015) measured FFR to a vowel-consonant-vowel stimulus. In addition to cortical sources (for 12 and 39 Hz), Herdman et al. (2002) modeled two brainstem sources, one vertically oriented and one laterally oriented. For the 88 Hz *f*_*m*_ (most similar to the rates in the current study), the main source dipole was in the brainstem. Bidelman (2015) found that FFR at the forehead followed the fundamental frequency of the speech but lacked a response to the higher harmonics, whereas FFR at the mastoid followed the fundamental and harmonics up to 1100 Hz. However, dipole modeling suggested a main source of FFR in the upper brainstem, oriented obliquely anterior to the vertex and parallel to the brainstem. While more caudal, horizontally aligned, sources contributed, they weighed less heavily on the overall response (Bidelman 2015).

In terms of the current study, the extent to which multiple sources influence the group delay estimate is dependent on whether the addition or subtraction waveform is used, and possibly on which montage is considered. For example, the CM is likely to have a greater influence on the group delay derived from the subtraction waveforms than on that derived from the addition waveforms. Because the CM is not half-wave rectified, addition should cancel out the CM and subtraction should enhance it (Picton et al. 1974; Sohmer and Pratt 1977). However, Chimento and Schreiner (1990) demonstrated that the addition of responses to alternating polarity stimuli (enhancing the envelope-FFR) will not always completely remove CM contamination. Therefore, one cannot assume that the addition waveforms contain no influence of the CM on the envelope-FFR group delay, but it is likely that the CM had a greater influence on the group delays derived from the subtraction waveforms (the TFS-FFR). Sohmer and Pratt (1977) and Davis and Britt (1984) suggested that very short latency (around 1 ms) responses are likely to be the CM. Stillman et al. (1978) found a CM (0.7 ms latency) in addition to the two neural FFRs they described. In the current study, group delay from the subtraction waveforms was generally longer than 3 ms, but there were three cases that may strongly represent CM (1.4 and 0.6 ms in the horizontal lower side-tone TFS-FFR, and 1.6 ms in the vertical higher side-tone TFS-FFR). The extent to which CM influenced each listener’s subtraction waveform FFR is unclear. It would depend on the relative strength and latency of the CM and the FFR. It would be useful, in future studies, to attempt to separate the CM from the FFR. One possible method of doing this is by measuring the effects of forwarding masking on the FFR (Chimento and Schreiner 1990). Whereas neural responses can be masked by a brief tone immediately preceding the probe tone, the CM is unaffected. This way pure CM signals can be recorded and subtracted from the mix of FFR and CM to derive a pure FFR signal.

Another possible contaminant of the horizontal montage TFS-FFRs could come from mechanical vibrations of the reference electrodes due to bone conduction to the mastoids (Small and Stapells 2004). This could create small microphonic artifacts; however, Small and Stapells (2004) only tested this at very high air-conduction stimulus levels (114 and 120 dB HL). In the current study, the extent of transduction of the acoustic stimuli into vibration at the mastoid is unknown.

## Summary

The results presented here support the assertion that a horizontal electrode montage records TFS-FFR from an earlier stage of the auditory pathway than does a vertical electrode montage. However, there was no evidence that envelope-FFR recorded by the two montages represents activity at different stages of the pathway. For both montages, the results suggest contributions from more rostral generation sites for the envelope-FFR than for the TFS-FFR. The results are consistent with previous reports of vertical and horizontal montages recording activity from different generators and suggest that group delay can provide a measure of latency for these generators.
